# Synthesis and properties of calix[4]arene telluropodant ethers as Ag^+^ selective sensors and Ag^+^, Hg^2+^ extractants

**DOI:** 10.3762/bjoc.5.59

**Published:** 2009-10-28

**Authors:** Yang Lu, Yuanyuan Li, Song He, Yan Lu, Changying Liu, Xianshun Zeng, Langxing Chen

**Affiliations:** 1 Key Laboratory of Display Materials and Photoelectric Devices (Tianjin University of Technology), Ministry of Education, Tianjin 300384, P.R. China; 2 State Key Laboratory of Elemento-Organic Chemistry, Nankai University, Tianjin 300071, P.R. China

**Keywords:** calix[4]arenes, extractants, mercury, sensors, silver, telluropodant ether

## Abstract

Three novel phenyltelluroalkoxyl functionalized tweezer-like calix[4]arenes **6**–**8** and two monophenyltelluropropoxyl functionalized calix[4]arenes **10** (cone conformer) and **12** (partial cone conformer) were synthesized and characterized. They are good Ag^+^-selective ionophores in ion-selective electrodes evaluated by electromotive force measurements of polymer membrane electrodes. The tweezer-like ionophores **6**–**8** showed excellent extraction ability towards Ag^+^ and Hg^2+^.

## Introduction

There is much interest in the development of compounds that selectively respond to specific metal ions for use as ion sensors. As the third generation of supramolecule, benefiting from their three-dimensional structures and easy chemical modification both at the upper- and lower-rims as well as their potential receptor properties for cations, anions and neutral molecules, calixarenes have enjoyed widespread use in various areas of science and technology. One of their successful applications as sensors is in analytical chemistry. They are useful for separations, enrichment, and analyses of ionic and neutral molecular species [1–4]. In particular, the ion-selective electrode (ISE) is an important target in analytical applications [5–10]. To improve the ion selectivity of calixarenes, a great deal of effort has been devoted to the design and synthesis of novel functionalized calixarenes in recent years [1–4]. In fact, a large number of calixarene derivatives containing pendant ether, amide, ketonic, ester and crown ether groups have been employed in studies of ISEs sensitive to sodium ions [11–20], potassium ions [21–25], caesium ions [26–30], thallium ions [31], lead ions [32–33] and organic ammonium ions [34–36]. But only a few reports are concerned with calixarenes as carriers sensitive to transition metal ions in the ionophore-based ISEs [30,37]. Due to their large covalent radius and greater polarizability compared to oxygen, the coordinating chemistry of heavier Group 16 analogues (S, Se, Te), especially sulfur, has attracted considerable interest in the chemical community. A number of ligands including pendant thioethers and crown thioethers with different denticity have been synthesized and their metal ion chemistry studied, producing a diverse range of structures and unprecedented electronic and redox response [38–49]. However, the use of the tellurium atoms as soft donors to design sensors and investigation of their ion selective performances have not been described to date. Aiming to construct receptor molecules which are sensitive to transition metal ions, a large number of calix[4]arene derivatives containing N, S, Se and P(III) atoms as soft donors have been prepared and their sensor properties have also been evaluated by ion-selective electrodes (ISEs) in our group and by our co-workers [50–53]. To continue our interest in the development of new ionophores, herein we describe the design and synthesis of three novel tweezer-like 25,27-dihydroxy-26,28-bis(phenyltelluroalkoxy)calix[4]arenes **6**–**8** composed of two tellurium atoms on the lower rims linked via methylene groups respectively, and study their Ag^+^-selective behavior by electromotive force measurements of polymer membrane electrodes.

## Results and Discussion

### Synthesis and conformations of calix[4]arene **6**–**8**, **10** and **12**

**Scheme 1:**
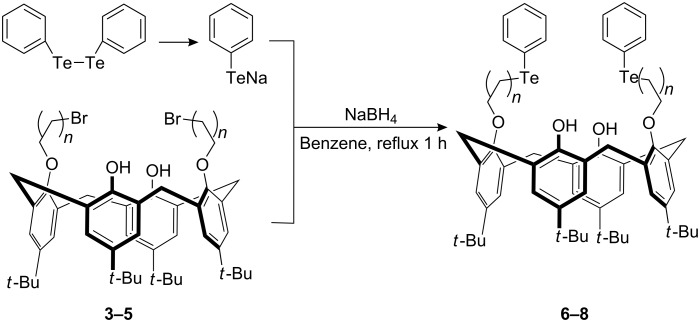
Synthesis and structures of calix[4]arenes **6**–**8**; **3**, **6**: n = 2; **4**, **7**: n = 3; **5**, **8**: n = 5.

As shown in Scheme 1, the calix[4]arenes **6**–**8** were synthesized in yields between 92% and 97% by the reaction of the preorganized cone conformation calix[4]arene dibromides **3**–**5** [54–55] with the sodium salt of phenyltellurate, which was prepared in situ by the reaction of diphenylditelluride with sodium borohydride in the presence of NaOH at the reflux temperature of ethanol–benzene (1:1, v/v). The structure and conformation of compounds **6**–**8** could be conveniently determined by their proton magnetic resonance spectra. As seen from ^1^H NMR data of **6**–**8**, the methylene bridge protons of the calix skeleton appeared as two doublets at nearly 4.25 ppm and 3.27 ppm and the signals of the calix aromatic protons and the upper-rim *tert*-butyl protons were separated as two singlet peaks in a 1/1 integration ratio, which all indicated that they are in cone conformation [56–57]. The methylene protons in the pendant region gave rise to different chemical shifts depending on the distance from O atoms or Te atoms, for example, those attached to the O atom appeared at 4.02–3.94 ppm and those close to the Te atom were found at 3.38–2.98 ppm. In addition, ^13^C NMR spectrum afforded some information about the influence of tellurium: the phenyl carbons attached to Te atoms were recorded at 111.79–111.57 ppm, while the signals of saturated carbons attached to Te atoms were detected at 8.60–4.88 ppm.

**Scheme 2:**
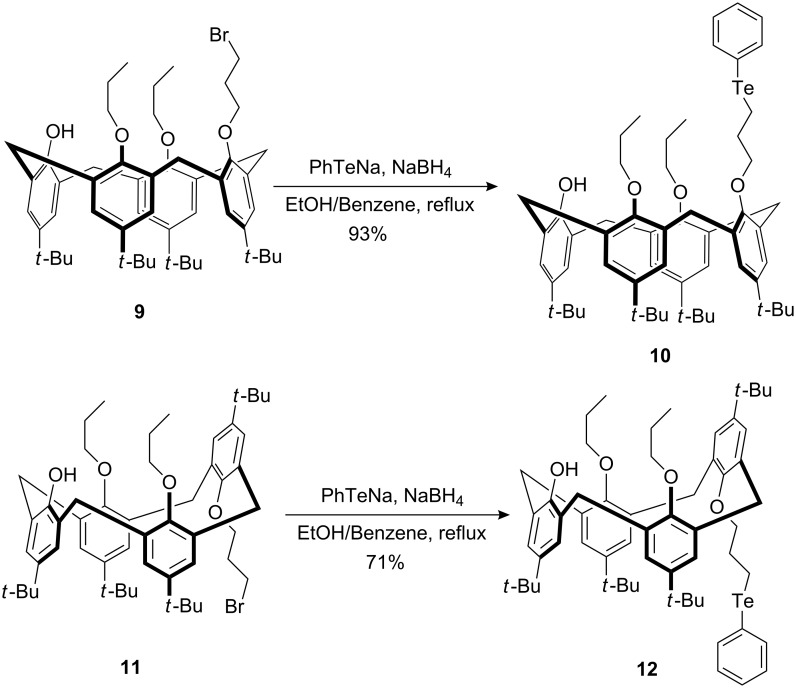
Synthesis of calix[4]arene **10** and **12**.

The cone and partial cone conformers of calix[4]arenes **10** (cone) and **12** (partial cone) were synthesized by the reaction of the cone and partial cone conformers of calix[4]arenes **9** and **11** with the sodium salt of phenyl tellurate in 93% and 71% yields, respectively (Scheme 2). Their structures were determined by ^1^H NMR and MS spectra. The phenyl carbon resonances attached to tellurium atoms were found at 112.08 ppm and 112.04 ppm, respectively. The signals of saturated carbons attached to tellurium were detected at 4.06 ppm and 3.54 ppm, respectively.

### Ion selectivity

For assessment of Ag^+^ ion selective behaviour of phenyltelluroalkoxyl modified calix[4]arenes derivatives (**6**–**8**, **10** and **12**), ISEs based on **6**–**8**, **10** and **12** as ionophores were prepared and their selectivity coefficients for Ag^+^ cations were investigated against alkali metal, alkaline-earth metal, lead, ammonium ions and some transition metal ions using the fixed interference method (FIM) [54–55]. Firstly, the response characteristics of Ag^+^-ISEs based on different ionophores were tested. It was noted that the Nernstian slopes of **6**–**8**, **10** and **12** based ISEs were between 49.4 ± 3.6 and 55.4 ± 1.3 mV·decade^−1^ to the activity of Ag^+^ ion within the activity range 10^−5.3^–10^−2.0^ M AgNO_3_, which indicated that all of calix[4]arenes **6**–**8**, **10** and **12** were good ionophores for Ag^+^-ISEs.

Furthermore, the Ag^+^ selectivity of phenyltelluride functionalized calix[4]arene **6**–**8**, **10** and **12** was evaluated by the potentiometric selectivity coefficients (log*K*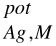) (Table 1). As shown in Table 1, the polymer membranes containing calix[4]arenes **6**–**8**, **10** and **12** as ionophores gave excellent log*K*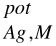 values (≤−3.3) against most of the interfering cations examined (i.e., Na^+^, K^+^, NH_4_^+^, Mg^2+^, Ca^2+^, Ni^2+^, Zn^2+^, Cu^2+^, Cd^2+^ and Pb^2+^), except that Hg^2+^ exhibited relatively lower discrimination (log*K*
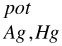
≤−1.4) compared with other interfering cations being examined. It is known that the smaller the log*K*
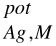
value, the greater the electrode preference for the primary ion over the interfering ions [54]. In other words, the lower value of selectivity coefficient obtained in present **6**–**8**-, **10**- and **12**-based ISEs illustrated their high Ag^+^ selectivity and only weakly response to the above interfering ions. The strong Hg^2+^ interference, once observed in some of ionophore-based ISEs [54] and traditional Ag_2_S-based [58–59] ISE is largely eliminated in the present of ISEs (log*K*
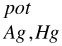
≤−1.4). It is reasoned that those ions with high hydration energies, such as Na^+^, K^+^, NH_4_^+^, Ca^2+^, Mg^2+^, Pb^2+^ and most of divalent transition metal ions, can not strongly interact with tellurium donors in the ionophores, while less heavily hydrated soft Ag^+^ ion could coordinate to soft tellurium donors selectively. Another interesting observation was that for **12**-based ISEs, the discrimination of Mg^2+^, Ca^2+^, Ni^2+^, Zn^2+^, Cu^2+^, Cd^2+^ and Pb^2+^ is almost one order of magnitude larger than those of **6**–**8**- and **10**-based ISEs. The weak binding ability of **12**-based ISEs towards Mg^2+^, Ca^2+^, Ni^2+^, Zn^2+^, Cu^2+^, Cd^2+^ and Pb^2+^ maybe explained by the fact that the monophenyltelluropropoxyl substituted group was inverted to the upper rim of **12** due to its partial cone conformation (Scheme 2), which made phenolic oxygen (once cooperating with tellurium atom to bind metal ions) difficult to interact with cations simultaneously. Although the calix aromatic π system was recognized as an assistant donor instead of phenolic oxygen atoms to bind cations [60–64], their binding ability towards metal ions unavoidably decreased in this experiment. Therefore, calix[4]arene **12** exhibited higher Ag^+^ selectivity due to its lower binding ability mainly towards Mg^2+^, Ca^2+^, Ni^2+^, Zn^2+^, Cu^2+^, Cd^2+^ and Pb^2+^.

**Table 1:** Selectivity coefficients (log*K*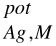) of the electrodes based on ionophores **6**–**8**, **10** and **12**.^a^

ion	log*K* 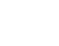				
	**6**	**7**	**8**	**10**	**12**

Ag^+^	0	0	0	0	0
Na^+^	−4.0	−4.0	−3.7	−3.8	−4.2
K^+^	−3.9	−3.6	−3.5	−3.8	−4.1
NH_4_^+^	−3.7	−4.1	−4.2	−3.5	−4.3
Mg^2+^	−4.5	−4.4	−4.3	−4.2	−5.2
Ca^2+^	−4.4	−4.2	−4.5	−4.5	−5.2
Zn^2+^	−4.1	−4.1	−4.1	−3.6	−5.1
Cu^2+^	−3.8	−3.9	−3.8	−3.3	−5.3
Ni^2+^	−4.1	−3.8	−4.0	−3.8	−5.3
Cd^2+^	−4.4	−3.9	−4.0	−3.8	−5.0
Pb^2+^	−4.0	−3.9	−4.0	−3.7	−4.9
Hg^2+^	−1.5	−1.8	−1.5	−1.4	−1.8

^a^The representative electrochemical cell for the EMF measurement was as follows: Ag·AgCl | int. soln. (0.01 M KCl) | PVC membrane | sample | salt bridge (3 M KCl) | saturated KCl | Hg_2_Cl_2_·Hg.

### Extraction behaviors

The extraction ability of tweezer-like receptors **6**–**8** for alkali, alkaline earth metals, lead, ammonium and some of transition metal cations was measured by Pederson’s method. The data are summarized in Table 2. For comparison, the cone and partial cone conformers of calix[4]arenes **10** and **12** were examined under the same conditions. As can be seen from Table 2, most of the receptors showed very weak extraction ability towards Li^+^, Na^+^, K^+^, Ca^2+^, which meant that these ionophores had weak affinity for these main Group cations. It was noteworthy that the soft Ag^+^ and Hg^2+^ ions were almost quantitatively extracted by calix[4]arenes **6**–**8**. The cone conformer **10** exhibited strong extraction ability towards Hg^2+^ and moderate extraction ability towards Ag^+^. However, the partial cone conformer **12** just gave moderate extraction ability towards Hg^2+^ and weak extraction ability towards Ag^+^. Thus, the extraction ability of the receptor **6**–**8**, **10** and **12** towards Ag^+^ and Hg^2+^ was given in the following order: Te_2_ (**6**–**8**) > Te (**10**, cone conformer) > Te (**12**, partial cone conformer). This sequence revealed that the soft tellurium donors play a key role in the binding with the soft Ag^+^ and Hg^2+^ cations and the phenolic oxygen atoms on the lower-rim may involve in the complexation with Ag^+^ and Hg^2+^ as assistant donors. These observations further proved that the cone conformation of calix[4]arene was much more favorable molecular framework for binding metal ions than corresponding partial cone conformation. In other transition metal ions cases such as Ni^2+^, Zn^2+^, Mn^2+^, Cu^2+^ and Cd^2+^, the receptors **6**–**8** and cone conformer **10** showed weak to moderate extraction ability (extraction% = 16.8–43.7%). But the extraction ability of partial cone conformer **12** towards these ions is much weaker than those of receptors **6**–**8** and **10**. Based on the better performance of receptor **10** compared with corresponding analogue **12**, it was further confirmed that the binding ability of phenolic oxygen donors on the lower rim of ligand **10** towards Ni^2+^, Zn^2+^, Mn^2+^, Cu^2+^ and Cd^2+^ was much stronger than those of the calix aromatic π system of ionophore **12**, which is in accordance with the complexation behavior in the ISEs though the solvent systems are significantly different.

**Table 2:** The percentage of metal picrates extracted from the aqueous to the organic phase by calixarenes **6**–**8**, **10**, and **12**.

ion	Extraction percentage (%)				
	**6**	**7**	**8**	**10**	**12**

Li^+^	12.8	11.8	1.7	1.1	2.3
Na^+^	0.9	0.9	0.7	0.5	5.4
K^+^	0	1.0	0	0.5	0.5
Ca^2+^	5.8	3.9	4.1	0.8	1.2
Cu^2+^	25.1	18.6	19.0	16.6	11.8
Mn^2+^	29.9	34.7	20.1	19.5	7.8
Cd^2+^	23.7	16.8	20.0	34.6	7.9
Ni^2+^	33.3	43.5	30.8	28.7	20.6
Zn^2+^	42.9	39.3	35.5	27.5	17.0
Pb^2+^	25.7	18.4	28.6	8.7	9.2
Hg^2+^	100	89.8	98.6	86.4	51.8
Ag^+^	100	100	100	68.3	21.2

## Conclusion

In summary, three novel phenyltelluroalkoxyl functionalized tweezers-like calix[4]arenes **6**–**8** and two monophenyltelluropropoxyl functionalized calix[4]arenes **10** (cone conformer) and **12** (partial cone conformer) were synthesized and characterized. Potentiometric selectivity evaluation showed that they are good Ag^+^-selective ionophores in ISEs. The tweezer-like ionophores **6**–**8** showed excellent extraction ability towards Ag^+^ and Hg^2+^. Their structure–selectivity relationships and comparative experiments with other ionophores containing S and Se donors are now being investigated and will be reported in due course.

## Experimental

### General Remarks

Melting points were determined with a Boetius Block apparatus. ^1^H NMR spectra were recorded on a Bruker AC-P200 spectrometer at 200 MHz in CDCl_3_ solution, using tetramethylsilane as an internal standard. ^13^C NMR spectra were recorded on a Bruker AC-P200 spectrometer at 50 MHz in CDCl_3_ solution. Elemental analyses were performed on a Perkin-Elmer 2400C instrument. Mass spectra were recorded on a VG ZAB-HS spectrometer. Compounds **3**–**5** [54–55] and **9** and **11** [50–53] were prepared according to literature procedures.

### 25,27-Dihydroxy-26,28-bis(phenyltelluropropoxy)-5,11,17,23-tetra-*tert*-butylcalix[4]arene **6**

Diphenyl ditelluride (512 mg, 1.25 mmol), prepared from phenyl Grignard reagent with tellurium powder in 78% yield, was dissolved in ethanol (30 ml) and benzene (30 ml) in a 100 ml round-bottomed flask. Under an atmosphere of nitrogen, solid sodium borohydride (228 mg, 6 mmol) was added in small portions to the solution until the orange color of the diphenyl ditelluride disappeared. The rest of the sodium borohydride was added in one portion. The colorless solution was heated to reflux. A solution of calix[4]arene dibromide **3** (1 mmol) in benzene (20 ml) was added. The reaction mixture was heated at reflux for 1 h, cooled to room temperature, and poured into water (100 ml). The mixture was extracted three times with chloroform. The combined extracts were washed thoroughly with water and dried over anhydrous sodium sulfate. The dry solution was filtered. The filtrate was evaporated to dryness under vacuum. The oily residue was purified by chromatography on silica gel with (CH_2_Cl_2_/petroleum ether v/v, 1/3) to give **6** as a white powder in 97% yield. FAB^+^-MS m/z 1139.9 (M^+^, Calcd, 1139.8). ^1^H NMR: 7.75 (d, 4H, *J* = 6.6 Hz, Te–Ph–H), 7.70 (s, 2H, OH), 7.23–7.16 (m, 6H, Te–Ph–H), 7.02 (s, 4H, Ar–H), 6.82 (s, 4H, Ar–H), 4.24 (d, 4H, *J* = 12.8 Hz, ArCH_2_Ar), 4.02 (t, 4H, *J* = 5.7 Hz, OCH_2_CH_2_), 3.38 (t, 4H, *J* = 6.4 Hz, TeCH_2_CH_2_), 3.29 (d, 4H, *J* = 12.8 Hz, ArCH_2_Ar), 2.34 (m, 4H, CH_2_), 1.26 (s, 18H, *t*-Bu–H), 0.98 (s, 18H, *t*-Bu–H). ^13^C NMR: 150.66, 149.49, 146.91, 141.38, 138.30, 132.63, 129.12, 127.59, 127.47, 125.53, 125.07, 111.62, 76.82, 33.87, 33.77, 31.97, 31.69, 31.00, 4.88. Anal. Calcd. for C_62_H_76_O_4_Te_2_: C, 65.30; H, 6.72. Found: C, 65.22; H, 6.79.

### 25,27-Dihydroxy-26,28-bis(phenyltellurobutoxy)-5,11,17,23-tetra-*tert*-butylcalix[4]arene **7**

Compound **7** was synthesized as yellowish oil in 96% yield. FAB^+^-MS m/z 1167.6 (M^+^, Calcd, 1167.8). ^1^H NMR: 7.75 (d, 4H, *J* = 6.2 Hz, Te–Ph–H), 7.56 (s, 2H, OH), 7.23–7.11 (m, 6H, Te–Ph–H) 7.02 (s, 4H, Ar–H), 6.79 (s, 4H, Ar–H), 4.21 (d, 4H, *J* = 13.0 Hz, ArCH_2_Ar), 3.94 (t, 4H, *J* = 5.7 Hz, OCH_2_CH_2_), 3.27 (d, 4H, *J* = 13.0 Hz, ArCH_2_Ar), 3.07 (t, 4H, *J* = 6.8 Hz, TeCH_2_CH_2_), 2.07 (m, 8H, CH_2_CH_2_), 1.27 (s, 18H, *t*-Bu–H), 0.96 (s, 18H, *t*-Bu–H). ^13^C NMR: 150.60, 149.73, 146.59, 141.16, 138.28, 132.44, 128.99, 127.59, 127.34, 125.32, 124.88, 111.57, 75.47, 33.74, 32.03, 31.60, 30.89, 28.32, 8.30. Calcd. for C_64_H_80_O_4_Te_2_: C, 65.78; H, 6.90. Found: C, 66.01; H, 6.79.

### 25,27-Dihydroxy-26,28-bis(phenyltellurohexoxy)-5,11,17,23-tetra-*tert*-butylcalix[4]arene **8**

Compound **8** was synthesized as yellowish oil in 92% yield. FAB^+^-MS m/z 1223.7 (M^+^, Calcd, 1223.9). ^1^H NMR: 8.10 (s, 2H, OH), 7.73 (d, 2H, *J* = 6.3 Hz, Te–Ph–H), 7.71 (d, 2H, *J* = 6.3 Hz, Te–Ph–H), 7.58 (m, 3H, Te–Ph–H), 7.18 (m, 3H, Te–Ph–H), 7.02 (s, 4H, Ar–H), 6.81 (s, 4H, Ar–H), 4.25 (d, 4H, *J* = 12.8 Hz, ArCH_2_Ar), 3.98 (t, 4H, *J* = 6.2 Hz, OCH_2_CH_2_), 3.27 (d, 4H, *J* = 12.8 Hz, ArCH_2_Ar), 2.98 (m, 4H, TeCH_2_CH_2_), 1.98–1.55 (m, 16H, –(CH_2_)_4_–), 1.31 (s, 18H, *t*-Bu–H), 0.97 (s, 18H, *t*-Bu–H). ^13^C NMR: 150.73, 149.67, 146.57, 141.19, 138.12, 132.61, 129.02, 127.76, 127.32, 125.39, 124.98, 111.79, 76.20, 33.77, 31.69, 30.99, 29.79, 25.23, 8.60. Calcd. for C_68_H_88_O_4_Te_2_: C, 66.69; H, 7.24. Found: C, 66.59; H, 7.28.

### General procedure for the synthesis of ditellurocalix[4]arenes **10** (cone) and **12** (partial cone)

Diphenyl ditelluride (128 mg, 0.31 mmol) was dissolved in ethanol (10 ml) and benzene (20 ml) in a 50 ml round-bottomed flask. Under an atmosphere of nitrogen, solid sodium borohydride (57 mg, 1.5 mmol) was added in one portion. The orange solution was heated to reflux for one hour. Then, a solution of calix[4]arene monobromide (214 mg, 0.25 mmol) in benzene (5 ml) is added. The reaction mixture was heated at reflux for 1 h, cooled to room temperature, and poured into water (40 ml). The mixture was extracted with chloroform for three times. The combined extracts were washed thoroughly with water and then dried over anhydrous sodium sulfate. The dry solution was filtered. The filtrate was evaporated to dryness under vacuum. The oily residue was purified by column chromatography.

### 25-Hydroxy-26,28-dipropoxy-27-(3-phenyltelluropropoxy)-5,11,17,23-tetra-*tert*-butyl-calix[4]arene **10** (cone)

Calix[4]arene **10** is obtained as yellowish oil in 93% yield (228 mg). FAB^+^-MS m/z 978.0 (M^+^). ^1^H NMR: 7.45 (d, 2H, *J* = 7.2 Hz, Te–Ph–H), 7.25–7.18 (m, 3H, Te–Ph–H), 7.15 (s, 2H, ArH), 7.02 (s, 2H, ArH), 6.51 (s, 2H, ArH), 6.46 (s, 2H, ArH), 4.18 (d, 2H, *J* = 12.9 Hz, ArCH_2_Ar), 4.16 (d, 2H, *J* = 12.9 Hz, ArCH_2_Ar), 4.05–3.75 (m, 6H, OCH_2_), 3.20 (d, 2H, *J* = 12.9 Hz, ArCH_2_Ar), 3.17 (d, 2H, *J* = 12.9 Hz, ArCH_2_Ar), 1.89–1.78 (m, 4H, CH_2_CH_2_Te), 1.33 (s, 9H, *t*-Bu–H), 1.31 (s, 18H, *t*-Bu–H), 0.97 (t, 6H, *J* = 9.1 Hz, CH_2_CH_3_), 0.79 (s, 9H, *t*-Bu–H). ^13^C NMR: 153.76, 151.64, 150.79, 145.73, 145.03, 141.30, 138.47, 135.97, 132.09, 131.82, 129.13, 127.40, 125.67, 124.98, 124.81, 124.66, 112.08, 75.82, 34.13, 33.81, 33.65, 31.74, 31.38, 31.07, 23.41, 10.76, 4.06. Calcd. for C_59_H_78_O_4_Te: C, 72.40; H, 8.03. Found: C, 72.45; H, 8.08.

### 25-Hydroxy-26,28-dipropoxy-27-(3-phenyltelluropropoxy)-5,11,17,23-tetra-*tert*-butyl-calix[4]arene **12** (partial cone)

Compound **12** is synthesized as yellowish oil in 71% yield. FAB^+^-MS m/z 978.2 (M^+^). ^1^H NMR: 7.65 (d, 2H, *J* = 7.4 Hz, Te–Ph–H), 7.45 (s, 1H, OH), 7.30–7.22 (m, 3H, Te–Ph–H), 7.07 (s, 2H, Ar–H), 7.04 (s, 2H, Ar–H), 6.90 (s, 4H, Ar–H), 4.16 (d, 2H, *J* = 12.5 Hz, ArCH_2_Ar), 3.94–3.88 (m, 4H, OCH_2_), 3.83 (s, 4H, ArCH_2_Ar), 3.22 (d, 2H, *J* = 12.5 Hz, ArCH_2_Ar), 3.01 (t, 2H, *J* = 7.3 Hz, OCH_2_), 2.45 (t, 2H, *J* = 7.3 Hz, OCH_2_), 1.97–1.52 (m, 6H, CH_2_), 1.43 (s, 9H, *t*-Bu–H), 1.29 (s, 9H, *t*-Bu–H), 1.11 (s, 18H, *t*-Bu–H), 1.03–0.92 (m, 6H, CH_3_). ^13^C NMR: 154.37, 152.69, 150.10, 145.32, 143.90, 141.47, 138.49, 133.54, 133.28, 132.75, 129.16, 128.81, 128.38, 127.47, 125.93, 125.09, 124.59, 112.04, 72.55, 70.48, 38.75, 33.95, 31.73, 31.55, 29.78, 23.21, 19.20, 10.49, 3.54. Calcd. for C_59_H_78_O_4_Te: C, 72.40; H, 8.03. Found: C, 72.66; H, 8.18.

**EMF Measurements.** All EMF (electromotive force) measurements were made at 25 °C, using a pH/mV meter. The sample solution was magnetically stirred and kept in a thermostatted water bath. The EMF values were corrected by subtracting the liquid-junction potential between the external reference electrode and the sample solution in the higher Ag^+^ concentration.

**Selectivity coefficients.** The potentiometric selectivity coefficient, *K*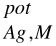, determined here is defined by the Nikolsky–Eisenman Equation (Equation 1).

[1]
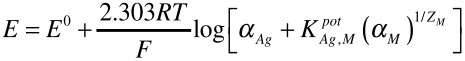


where *E* represents the experimentally observed potential, *R* the gas constant, *T* the thermodynamic temperature in *K*, *F* the Faraday constant, *a*_Ag_ the Ag^+^ activity, *a*_M_ the activity of the interfering cation, and *Z*_M_ the charge of the interfering cation. The selectivity coefficients were determined by a mixed-solution method [65–66]. In this mixed-solution method, the concentration of silver ion is varied while that of the interfering ions such as Na^+^, K^+^, NH_4_^+^, Ca^2+^, Mg^2+^ are 0.1 M; Zn^2+^, Cu^2+^, Ni^2+^, Cd^2+^, Pb^2+^, Hg^2+^ are 0.01 M. According to this method, the potentiometric selectivity coefficients, *K*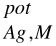, can be evaluated from the potential measurements on solutions containing a fixed concentration of the interfering ions (M^n+^) and varying the concentration of Ag^+^ ion using Equation 2.

[2]
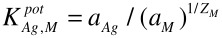


The resulting log*K*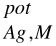 values are summarized in Table 1.

**Extraction experiments.** Extraction experiments were carried out using a solution of metal picrate in water saturated with dichloromethane (1.00 × 10^−4^ mol·L^−1^) and solutions of the ligand in water saturated with dichloromethane (1.00 × 10^−3^ mol·L^−1^). Equal volumes (0.01 L) of the mutually saturated solvents containing the metal ion salt in the aqueous phase and the ligand in the organic phase were shaken for 30 min by 2D-2 oscillator and then left for 2 h at (25 ± 0.05) °C. For the determination of picrates in water phase, a cary-300 UV-visible spectrophotometer was used and absorbance readings were taken at 354 nm.
